# The Effect of Neuromuscular Electrical Nerve Stimulation in the Management of Post-stroke Spasticity: A Scoping Review

**DOI:** 10.7759/cureus.32001

**Published:** 2022-11-29

**Authors:** Athanasios Chasiotis, Vasileios Giannopapas, Marianna Papadopoulou, Maria Chondrogianni, Dimitrios Stasinopoulos, Sotirios Giannopoulos, Daphne Bakalidou

**Affiliations:** 1 Department of Physiotherapy, University of West Attica, Athens, GRC; 2 Department of Physical Therapy, University of West Attica, Athens, GRC; 3 2nd Department of Neurology, Attikon University Hospital, Athens, GRC; 4 Physiotherapy, University of West Attica, Athens, GRC; 5 Laboratory of Neuromuscular and Cardiovascular Study of Motion (LANECASM) Department of Physiotherapy, Faculty of Health and Care Sciences, University of West Attica, Athens, GRC

**Keywords:** acute hemiplegia, physical therapy rehabilitation, spasticity, stroke, neuromuscular electrical stimulation

## Abstract

Stroke is a cerebrovascular disorder characterized by the sudden onset of symptoms and clinical signs caused by either vascular infraction or hemorrhage. One of the main symptoms in the majority of post-stroke patients is spasticity. The main therapeutic options of spasticity in post-stroke patients include pharmacological interventions, rehabilitation techniques, and surgery. This review aims to explore the effectiveness of Neuromuscular Electrical Stimulation (NMES) for post-stroke spastic hemiparetic limb (upper and lower). Thorough research of the PubMed Medline database was performed. Records were limited to clinical studies published between 01/01/2010 and 01/01/2022. The results were screened by the authors in pairs. The search identified 26 records. After screening, nine records met the inclusion-exclusion criteria and were assessed. There were seven studies for spastic upper limbs and two for spastic lower limbs. The approaches investigated the effectiveness of electrical stimulation on post-stroke spastic upper or lower limb. Spasticity was measured through the modified Ashworth scale (MAS) and electromyographic recordings (EMG). In most cases, spasticity was decreased for at least two weeks post-intervention. In conclusion, NMES can be used either solo or in combination with different physical therapy modalities in order to produce optimal results, taking into consideration the specific needs and limitations of each individual patient. Based on the existing literature, as well as the limitations of the included studies, the authors believe that future studies on the subject of NMES in the management of post-stroke spasticity should focus on carefully examining each electrical parameter.

## Introduction and background

Stroke is a cerebrovascular disorder characterized by the sudden onset of symptoms and clinical signs caused by either vascular infarction or hemorrhage [1]. It is considered one of the leading causes of death due to cardiovascular disease (CVD) worldwide, reaching a mortality rate of 33% of CVD patients in 2020 [1]. Simultaneously, the majority of strokes (ischemics and hemorrhagics) lead to long-term disability affecting the patient’s motor and sensory function, cognitive status, bladder, bowel, and sexual function [2].

One of the main symptoms in the majority of post-stroke patients (pSps) is spastisticity, a motor disorder characterized by a velocity-dependent increase in tonic stretch reflexes with exaggerated tendon jerks, which is a typical sign of upper motor-neuron syndrome (UMNS). Spasticity is considered a “positive” feature of UMNS due to the loss of inhibition of the lower motor neuron pathways resulting from a sensory-motion control disorder in the muscle regulation system. PSps usually present with an eclectic, lateralized sensory and motor disorder, with their affected upper limb exhibiting a hypertonic flexion pattern while the equilateral lower limb exhibits a hypertonic extension pattern [2].

With one out of five first-ever stroke patients developing spasticity, many studies have examined and demonstrated its negative impact on the quality of life (QoL) of pSps due to the reduction of the mobility and functional use of the affected limbs, which restricts the person from working, performing daily life activities (ADL) and socializing. In addition, a significant percent of pSps exhibits a high degree of disability, thus needing around-the-clock assistance from a caregiver (usually a family member) [2].

The main therapeutic options of spasticity in post-stroke patients include botulinum toxin intramuscular injections, baclofen (per os or via intrathecal pump), intraneural phenol injections, surgical procedures aiming at altering the muscular, neural, or tendon structures, and physical therapy, which consists of stretching and strengthening exercises, hydrotherapy, and electrical stimulation (ES) [3].

Electrical stimulation is a supplementary modality with a variety of types that is utilized to increase muscle strength, reduce pain, and reduce hypertonia in the affected limbs [3,4]. Neuromuscular nerve stimulation (NMES) is a specific type of electrical stimulation that is used to produce muscle contractions through the application of an electrical stimulus in the distal part of a specific nerve [3,5]. Since the electrical excitability of lower motor units (and their respective innervated muscles) is usually intact, NMES can be used to stimulate the neuromuscular activity of the affected limbs with either direct stimulation of the affected muscles or the stimulation of their antagonists solo or in parallel with robotic assistive devices [6,7].

The purpose of this scoping review is to investigate the effectiveness of NMES in increasing the mobility and/or functionality of the affected upper and lower limb in pSps.

Methodology

**Figure 1:**
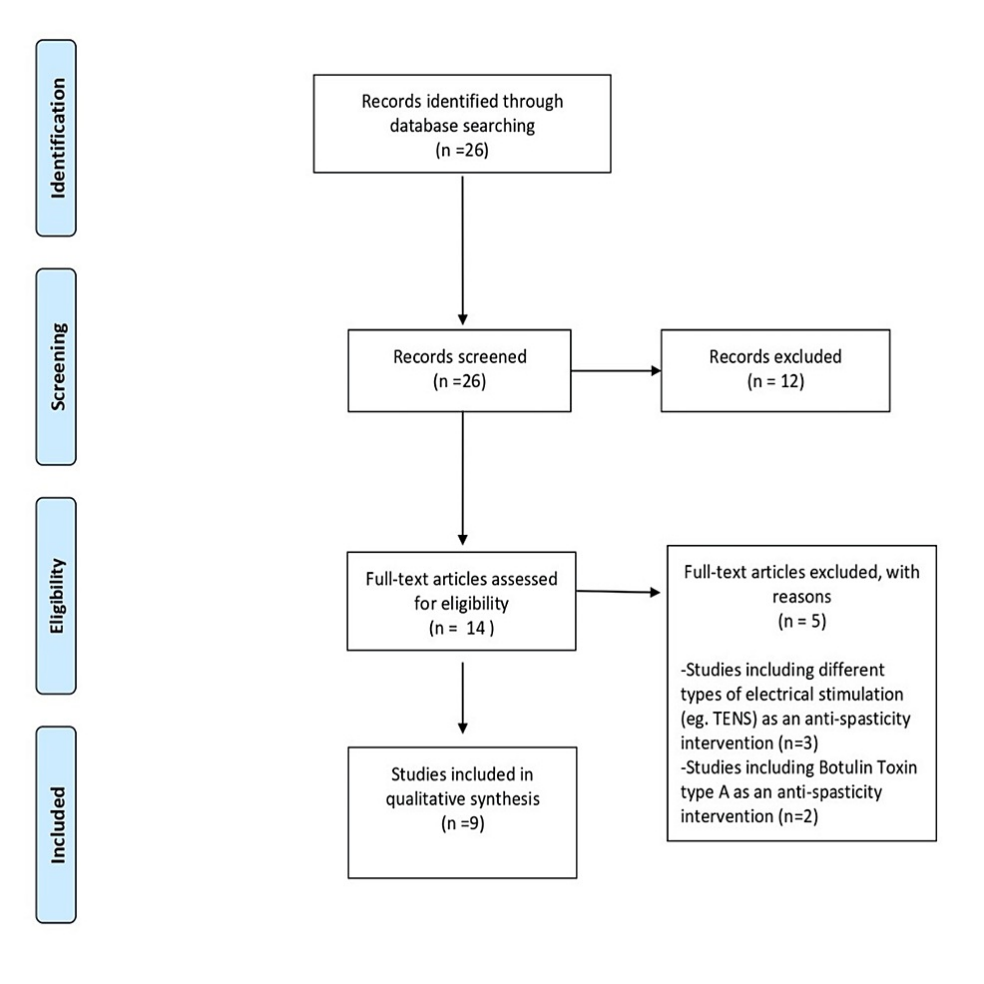
PRISMA flowchart PRISMA=Preferred Reporting Items for Systematic Reviews and Meta-Analyses

A scoping review of clinical trials and randomized control trials regarding the use and effectiveness of NMES in the mobility and/or functionality of the affected upper and/or lower limb in pSps was performed according to the Preferred Reporting Items for Systematic Reviews and Meta-Analyses (PRISMA) guidelines [8]. Three authors independently performed the literature search and assessed the results before the synthesis. Studies reporting intervention with NMES on spastic upper or lower limbs were included while protocols presenting NMES treatments combined with physiotherapeutic and/or pharmacological techniques were excluded from the literature search. The studies included were based on spasticity and the thorough investigation of its therapeutic approach. Most included studies based their design and the sample stratification using the modified Ashworth scale (MAS), hence the database search was adjusted accordingly. A MESH search was performed using the PubMed Medline database with the terms: electrical neuromuscular stimulation AND stroke AND spasticity Filters: Clinical Trial, Randomized Controlled Trial, from 01-01- 2010 - 01-01-2022. The corresponding flowchart is presented in Figure 1 and the included studies are presented in Table 1.

**Table 1:** Table of studies *RCT=randomized controlled trial, WF=waveform, F=frequency, PD=pulse duration, I=intensity, AS=application site, TD=treatment duration, EMG=electromyography, APB=abductor pollicis brevis, ED=extensor digitorum, FMA=Fugl-Meyer assessment, MAS=modified Ashworth scale, ARAT=action research arm test, FIM=functional independence measure, FD=flexor digitorum, TRI=triceps brachii, BIC=biceps brachii, WMFT=Wolf motor function test, MAL=motor activity log, SIS=stroke impact scale 3.0, EC=extensor carpi, MBI=modified Barthel index, ECR=extensor carpi radialis, ECU=extensor carpi ulnaris, BS=Brunnstrom scale, ECC=extensor carpi communis, VRS=verbal rating scale, PROM=passive range of motion, (L+B)=longus and brevis, EDC=ED communis, MESUPES=motor evaluation scale for upper extremity in stroke patients, BBT=box and block test, BI=Barthel index, ACR=antagonist co-activation ratio, EC=extensor hallucis, CSS=composite spasticity scale, AADS=ankle active dorsiflexion score, TUGT=time up and go test, TA=tibialis anterior, MG=medial gastrocnemius

Study	Type of Study	Patients	Electrical Parameters	Outcome Measures	Results
Lin et al, 2011 [9]	Single-blinded RCT	37 stroke patients	WF: Symmetrical, biphasic. F: 30 Hz. PD: 300μsec. Ramp up/down: 1sec/1sec. ON/OFF time: 5sec/5sec. I: 0-90mA. AS: supraspinatus, deltoid. EC TD: 30min/session. 5 days/week, 3 weeks	MAS, FMA, MBI	*Decreased MAS for 3 months p<.05 increased fma after weeks
Sahin et al, 2012 [10]	RCT	50 stroke patients	WF: Pulsed, F: 100 Hz, PD: 0.1 msec (100μsec), Width between 2 pulses: 0.9msec (900μsec), Width between 2 cyclic pulses: 3 msec. Resting time: 9sec, AS: ECR, ECU. TD: 15min/session, 5 days/week, 1 month	MAS Goniometer EMG BS FIM	*Decreased MAS (p=.001). *Increased FIM (p=.028)
Boyaci et al, 2013 [11]	RCT	31 stroke patients	GROUP A: EMG-triggered NMES (active NMES). WF: Symmetrical, biphasic. F: 50Hz. PD: 200μsec. I: 20-47mA. Ramp up/down: 2sec/2sec. Sensitivity EMG biofeedback = 0-100μV. AS: ECC + ECU TD: 45 min/session, 5 days/week, 3 weeks. GROUP B: (passive NMES). WF: Symmetrical, Biphasic. F: 50Hz. PD: 200μsec. I: 20-47mA Ramp up/down: 2sec/2sec. Sensitivity EMG biofeedback = 0-100μV. AS: ECR + ECU. TD: 45min/session, 5 days/week, 3 weeks.	FMA, FIM, MAL, MAS, Goniometer, Jamar, Hand dynamometer, Surface EMG potentials	*Improved ROM & FMA in passive NMES (P<.05 significant changes on the other measurements)
Malhotra et al, 2013 [12]	RCT	90 stroke patients	WF:300 μsec. ON/OFF time: 15sec/15sec. Ramp Up/Down: 6sec/6sec. F: 40Hz I: maximum muscle contraction. AS: wrist+ finger extensors. TD: 30min, 2 times/day, 5 days/week, 6 weeks	VRS, Muscle activity test, PROM, ARAT	*Decreased VRS (p=.02), *No changes in spasticity & stiffness (p=.02).
Lee et al, 2015 [13]	Double-blind sham RCT	39 stroke patients	WF: Symmetrical, rectangular, biphasic F: 30Hz PD: 200μsec AS: FD-ED, Pronator-Supinator TD: 20-30min	FMA MAS WMFT MAL SIS	*Decreased MAS (p=.017). *No improvement in FMA, MAS, SIS and ADL (p=.017).
Wang et al, 2015 [14]	RCT	72 stroke patients	WF: symmetrical, biphasic, square. PD: 200μsec, F: 20Hz, ON/OFF time: 5sec/5sec, I: sensory threshold, motor threshold & full-movement, NMES TD: 30min, 2 times/day, 5 days/week, 4 weeks AS: EC + EDL	CSS AADS TUGT	*Decreased CSS in full-movement NMES (p<.05 aads in full-movement nmes differences tugt>.05).
Yang et al, 2018 [15]	RCT	25 stroke patients	WF: biphasic, square. F: 50Hz. PD: 200μsec. ON/OFF time: 5/15sec. I: 50-0mV (full ROM contraction). AS: TA, MG TD:20min, 3 times/week, 7 weeks	GAITRite system, MAS, EMG activity, Goniometer, Handheld dynamometer	*Decreased MAS in NMES-TA group in static (p=0.028) and dynamic spasticity (p=.025). *Increased muscle strength in NMES-TA group (p=0.009). *Increased ankle plantar flexion during push off in NMES-TA group (p=.015).
Huang et al, 2020 [6]	RCT	30 stroke patients	WF: Rectangular PD: 0-300μsec Pulse Width: 70V F: 40Hz TD: 3-5 sessions/week, 7 weeks AS: APB, ED	FMA MAS ARAT FIM EMG parameters (APB, ED, FD, TRI, BIC)	*Decreased MAS (p=.018) for 3 months (p<.05 fma flm).
Mano et al, 2021 [16]	RCT	61 stroke patients	WF: Symmetrical, rectangular. Biphasic F: 35Hz (1^st^ group) & 50Hz (2^nd^ group). PD:300μsec. I: maximum muscle contraction. Ramp Up/down: 2sec/2sec (the 1^st^ week) & 1sec/1sec (rest of the study). Contraction-relaxation time: 5-25sec (2 weeks), 5-20sec (3^rd ^week), 5-15sec (4^th^week), 5-10sec (5^th^, 6^th^ week) & 5-5sec (final weeks) TD: 20min (first 2 sessions) & 30min (rest sessions), 3days/week, 8weeks & 2-months follow-up. AS:ECR (L+B) + EDC	MESUPES-arm test subscale, Goniometer, EMG activity, MAS, BBT, BI	*Improved ROM in 35Hz and in 50Hz with greater results in 35Hz (p<0.01). *Improved grip strength in 35Hz and 50Hz with greater results in 35Hz (p=0.016). *Decreased MAS in 35Hz than 50Hz (p=0.002) *Larger EMG amplitude extensors in 35Hz (p>0.05) and ACR extensors in 50Hz (p>0.05). *Improved BI in 35Hz (p<0.01).

## Review

Results

There were a total of nine trials regarding the application of NMES in pSps spasticity [6,9-16]. All of them were non-pharmacological and interventional. Seven of them investigated the effectiveness of NMES on the spastic upper limb while two of them focused on the spastic lower limb [6,9-16]. The majority of the included studies utilized the MAS and/or electromyographic recordings in order to quantify the effect of NMES.

NMES Application on Upper Limb Spasticity

A study by Lin and colleagues examined the effectiveness of NMES on spastic carpal extensor muscles on 37 pSps who were randomly assigned to two groups, a 30 Hz, 300 μsec pulse duration (PD)-symmetrical biphasic waveform-NMES group (group A) and a control group. The intervention lasted for three weeks with follow-ups in one, three, and six months and were monitored using evaluation scales such as MAS and Fugl-Meyer Assessment (FMA). There was a significant decrease on the MAS score (p<.005) for the active group, which was maintained in the one-month follow-up [9]. Similar results regarding the management of post-stroke spasticity on carpal extensor muscles were presented by Sahin and colleagues using higher frequency NMES (100 Hz, 100 μsec pulsed (PD) waveform) in 50 pSps, which led to decreased spasticity for approximately one month post-intervention (p<.001) [10].

Mano and colleagues conducted a clinical trial in order to compare the effects of two NMES protocols with different stimulation frequencies on upper limb motor impairment in adults with spastic hemiparesis after stroke. Sixty-one patients were randomly assigned to the control group or the experimental groups (NMES with 35 Hz and 50 Hz, 300 μsec, and symmetrical rectangular biphasic waveform) for eight weeks. NMES was applied in extensor carpi radialis and extensor digitorum communis muscles. Outcome measures for spasticity included MAS and EMG activity. The authors concluded that the 35Hz NMES protocol decreased MAS scores (p=0.002) and had larger EMG amplitude in wrist extensors (p<.05) [16].

On the other hand, a study of Boyaci and colleagues investigated the efficacy of electromyography (EMG)-triggered NMES (50 Hz-200 μsec PD-symmetrical biphasic waveform, active range of movement (ROM) NMES), and passive NMES (50 Hz-200 μsec PD-symmetrical biphasic waveform, passive ROM NMES) in enhancing the upper extremity motor and functional recovery of 31 stroke patients who were randomly assigned into three groups: active-NMES, passive-NMES, and control groups. Each treatment was applied five times per week for three weeks. Patients were evaluated through FMA, functional independence measure (FIM), MAS, goniometric measurements, as well as surface EMG potentials. Their results showed improved ROM and FMA scores in passive NMES (p<.05), without any alterations in spasticity (p<.05) [11].

Malhotra and colleagues investigated the effects of surface NMES (40 Hz, 300 μsec PD, n/a waveform) on the extensor muscles of the wrist and upper limb. The intervention lasted six weeks with each session lasting 30 mins with a frequency of two sessions per day. Spasticity was evaluated using the muscle activity test and the passive range of motion (PROM) of wrist and fingers. Despite no statistical significant change in spasticity, the authors reported a significant decrease in pain levels (p=.02) [12].

Aside from the use of NMES as a solo intervention for upper limb spasticity management, two studies utilized NMES with robotic applications.

Lee and colleagues examined the effects of a 30 Hz, 200 μsec PD-symmetrical biphasic waveform electrical stimulation delivered by a robotic glove with integrated electrode focused on spastic muscles (flexor digitorum and pronator muscles) as well as on antagonist-to-spasticity muscles (extensor digitorum and supinator muscles). Their findings showed that NMES decreased spasticity levels on the spastic muscles (p=.017) [13]. In the same spirit, Huang and colleagues examined a 40 Hz, 0-300 μsec PD, rectangular waveform NMES protocol focused only on the antagonist-to-spasticity muscles for seven weeks. Their results showed that NMES decreased elbow, wrist, and finger MAS scores, which were persistent on the three-month follow-up (p<.05) [6].

NMES Application on Lower Limb Spasticity

In a mixed population RCT, Wang and colleagues examined the effects of 50 Hz, 200 μsec, symmetrical biphasic square NMES combined with full active ROM movement in the reduction of spasticity focusing on the plantar flexors. Patients received 30-minute sessions of NMES on the motor points of the extensor hallucis and digitorum longus twice a day, five days per week for four weeks. The results were evaluated using the composite spasticity scale (CSS), ankle active dorsiflexion score (AADS), and walking time in the timed up and go test (TUGT). The authors reported significant reduction in the CSS scores (p<.05) and improvement in AADS scores (p<.05), which maintained at the two-week follow-up [14].

Yang and colleagues assessed the efficacy of 50 Hz, 200 μsec PD, biphasic square waveform NMES on either the dorsi flexors or plantar flexors muscles during walking and gait performance in 25 stroke patients with inadequate ankle control. The participants were divided into three equal groups. The experimental group received 20 minutes of NMES either on the tibialis anterior muscle (NMES-TA) or the medial gastrocnemius muscle (NMES-MG) while the control group received 20 minutes of ROM and stretching exercises. The static and dynamic spasticity of ankle plantar flexors during gait was assessed using the MAS and EMG activity of the muscles and their lengthening velocities. The authors reported a statistically significant reduction in static (p=.028) and dynamic spasticity (p=.025) of the ankle plantar flexors of the NMES-TA group as well as improvements in muscle strength (p=.009) and active ankle plantar flexion during push off (p=.015) [15].

Discussion

NMES can be used either solo or in combination with different physical therapy modalities in order to produce optimal results, taking into consideration the specific needs and limitations of each individual patient.

The application of NMES on the dorsiflexor muscles of the hemiplegic lower limb for four weeks resulted in significant improvements on the FIM motor subscore, which translates to clinical improvement of the post-stroke hemiplegic lower limb [17]. Furthermore NMES on the extensor hallucis and digitorum longus leads to improved plantar flexion while its application directly on top the motor points of the tibialis anterior muscle prompted the reduction in the static and dynamic spasticity of the plantar flexors and the improvement of plantar flexion during push-off [14,15].

In combination, NMES with ROM and/or stretching exercises can further reduce spasticity levels with the effects lasting one to three months post-intervention, depending on the parameters. Simultaneous application of NMES with neurodevelopmental techniques (e.g. Bobath) resulted in significant reduction of spasticity in the plantar flexors with a simultaneous increase of the ankles passive range of motion, which was maintained for approximately three months post-intervention while the most promising results were demonstrated by the use of NMES and robotic applications with a long-lasting significant reduction of spasticity in the elbow, wrist, and fingers of pSps [13,15,17,18].

It is worth noting that the application of high-frequency NMES in pSps can sometimes lead to muscle fatigue and reduction of contractile forces, which sequentially increases the affected limbs muscle tone [11,12]. On the other hand, even in cases where the use of NMES showed no improvement on the limb’s spasticity levels, there were significant improvements in pain perception [19].

Most of the included studies share two common limitations. First, the sample size was relatively small and did not allow any generalization of the results. Second, each study focused on one or two electrical parameters. In addition, the included studies selected different electrical parameters, which resulted in the absence of a homogenous NMES protocol for the management of post-stroke spasticity.

Based on the existing literature, as well as the limitations of the included studies, the authors believe that future studies on the subject of NMES in the management of post-stroke spasticity should focus on carefully examining each electrical parameter. More specifically, the existing literature presents some data regarding the differences between high and low frequency, different site applications, and the waveform of NMES in pSps rehabilitation.

In most neurological conditions, the frequency is set between 20 and 100 Hz in order to facilitate the recruitment of type I (20-35 Hz) and type II (30-70 Hz) fibers while frequencies above the 100 Hz threshold have been shown to lead to the development of rapid muscle fatigue in pSps [20,21]. The most commonly used waveform is symmetrical biphasic rectangular with some studies experimenting with either square type or pulse waveform. Finally, the range of pulse duration of the electrical stimulation is 200-450 μs (0.2-0.45 ms) with 350-450 μs being the most commonly used to activate hemiparetic muscles since lower pulse duration may be more comfortable for the patient but not as effective and pulse duration higher that 450 μs may increase the total electrical charge absorbed by the targeted structure depending on the application time. However, pulse duration higher than 450 μs may lead to more efficient activation of large diameter afferent nerve fibers, which in turn may lead to an increase in central motor-neuron recruitment [20,21].

## Conclusions

Neuromuscular electrical stimulation is a common therapeutic modality used by physical therapists in a wide range of musculoskeletal and neurological rehabilitation protocols including post-stroke rehabilitation. The aim of this systematic review was to examine the efficacy of NMES as a treatment modality in the management of post-stroke spasticity on the upper or lower hemiplegic limb and to identify the different electrical parameters (waveform, pulse duration, frequency, on/off time, site of application, intensity) that are being used. One of the main things highlighted by this systematic review is the heterogeneity of the electrical parameters. Even though there is a variety of possible NMES protocols for the management of post-stroke spasticity, there is an urgent need for a standarized, validated protocol. Based on the literature review and clinical experience, the frequency and waveform used for the management of post-stroke spasticity are commonly set in the specified range (0-100 Hz and biphasic symmetrical, respectively). Future studies should focus on examining the effect of a higher and/or lower pulse duration (specifically lower than 100 μs and higher than 450 μs) with a variation in the treatment duration in order to avoid any negative impact on pSps. Furthermore, it would be beneficial to maintain the homogeneity of all other electrical parameters and the heterogeneity of the sample in the domains of gender, age, and socio-economic status.
